# Congenital fibrosis of the extraocular muscles

**DOI:** 10.4103/0974-620X.64230

**Published:** 2010

**Authors:** Pascale Cooymans, Sana Al-Zuhaibi, Rana Al-Senawi, Anuradha Ganesh

**Affiliations:** Department of Ophthalmology, Sultan Qaboos University Hospital, Muscat, Oman

**Keywords:** CFEOM, ophthalmoplegia, ptosis, restrictive strabismus

## Abstract

**Background:**

Congenital fibrosis of the extraocular muscles (CFEOM) describes a group of rare congenital eye movement disorders that result from the dysfunction of all or part of the oculomotor (CN 3) and the trochlear (CN 4) nerves, and/or the muscles these nerves innervate.

**Aim:**

To describe the clinical and neuro-radiological findings in three patients with CFEOM and review literature with respect to clinical features, genetics and management of this condition.

**Materials and Methods:**

A retrospective chart review was performed of three Omani patients who had been diagnosed with CFEOM in our institution. All patients had undergone standardized orthoptic and ocular evaluations and magnetic resonance imaging (MRI) of the orbits and brain.

**Results:**

The three patients (age range nine months - 10 years) presented a history of congenital strabismus. All patients had severe bilateral ptosis and mild to moderate visual impairment secondary to the ptosis and astigmatism. Two of three patients demonstrated a positive jaw-winking phenomenon. A moderate to large angle exotropia with varying amount of hypotropia and limitations of almost all the extra ocular muscles was noted. Patient 3 was also developmentally delayed. MRI brain and orbit showed abnormalities of the extraocular muscles in two patients and brain malformation in one patient.

**Conclusions:**

CFEOM is a rare, congenital, and non-progressive disorder with multiple extra ocular muscle restrictions. CFEOM can be associated with neuro-radiological abnormalities; its diagnosis and classification is defined by clinical characteristics and genetics. Options for treatment are limited and difficult.

## Introduction

CFEOM is the term used to describe several different inherited strabismus syndromes which manifest as congenital restrictive ophthalmoplegia (restriction of globe movement in one or more fields of gaze), affecting extraocular muscles innervated by the CNIII and/or CNIV. The term Congenital Cranial Dysinnervation Disorders (CCDDs) was coined to refer to the innervation disorders of the extraocular muscles.[1] The various forms of CFEOM are included in the CCDDs.

In this paper we describe the clinical and neuro-radiological findings in three patients with CFEOM who presented to us with a history of congenital strabismus and ptosis, and review literature with respect to clinical features, genetics and management of this condition.

## Materials and Methods

A retrospective chart review was performed of three Omani patients who had been diagnosed with CFEOM in our institution between the period 2003–08. All patients had undergone standardized orthoptic and ocular evaluations. Magnetic resonance imaging (MRI) of the orbits and brain was performed in all patients as part of the evaluation of patients with CFEOM.[2]

This study was approved by the Departmental Research Committee.

## Results

Three Omani patients in the age range of nine months to 10 years were referred for evaluation and diagnosis of complex strabismus. The ocular deviation had been noted in all the three patients from birth. Patient 3 was also developmentally delayed. While patient 1 had undergone bilateral frontalis suspension for congenital ptosis, none of the others had received any treatment.

**Figure 1:**
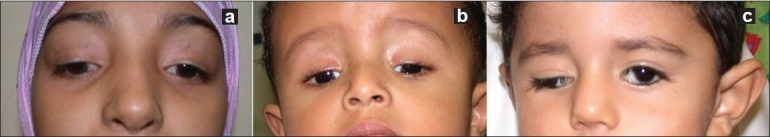
Photograph showing a) Patient 1, b) Patient 2, and c) Patient 3. Note complete to severe bilateral ptosis, moderate to large angle exotropia and hypotropia

**Figure 2:**
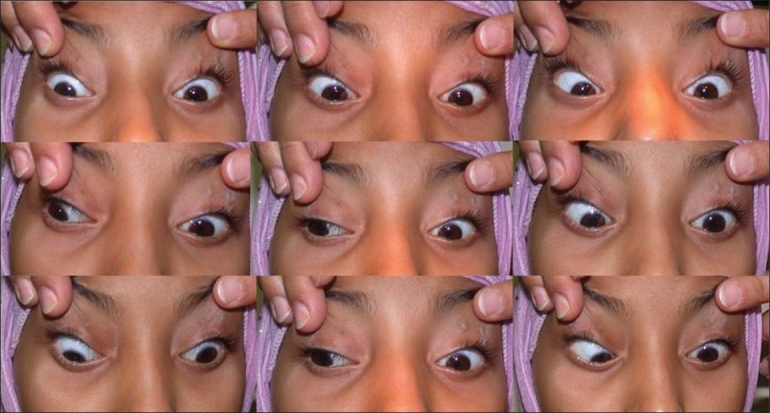
Ocular versions in Patient 1. There is restricted action of multiple extraocular muscles, with complete lack of vertical eye movements, relatively better horizontal movements, downshoot of the right eye in adduction, and limited abduction of the left eye. The non-absorbable sutures used for frontalis suspension are clearly visible in both eyelids

**Figure 3:**
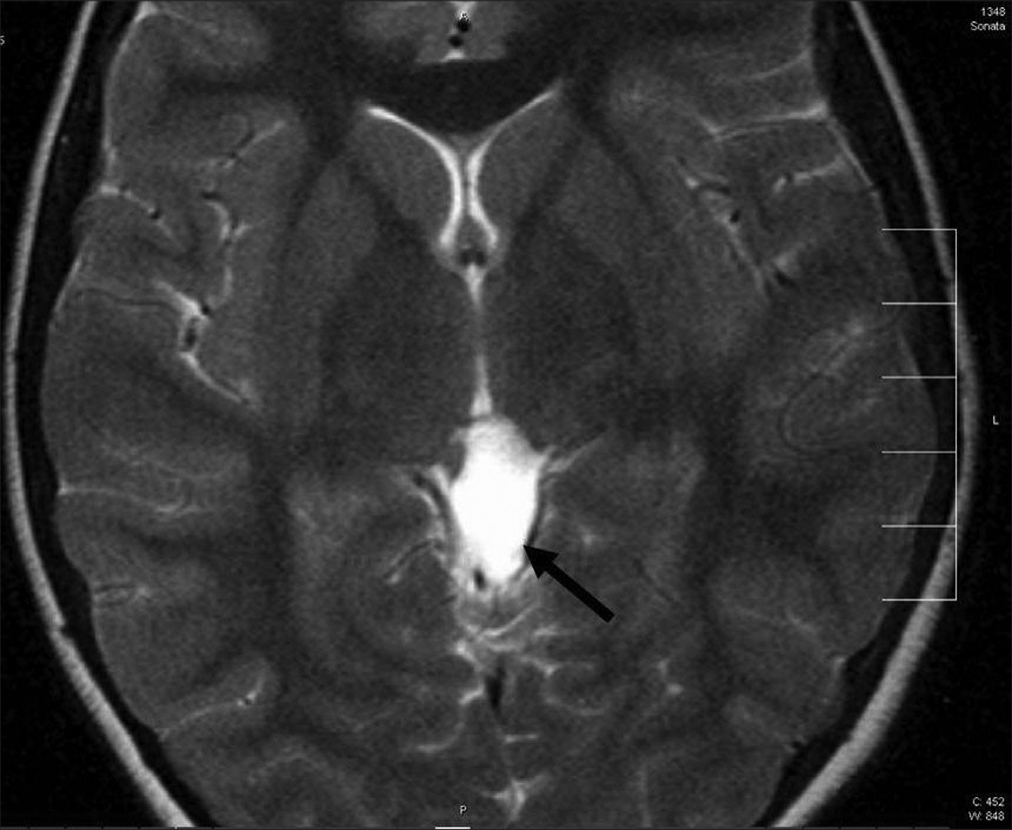
MRI brain of patient 1. T2-weighted image showing a mass in quadrigeminal cistern (arrow), isointense to cerebrospinal fluid, likely an arachnoid cyst, with compression of quadrigeminal plate

**Figure 4:**
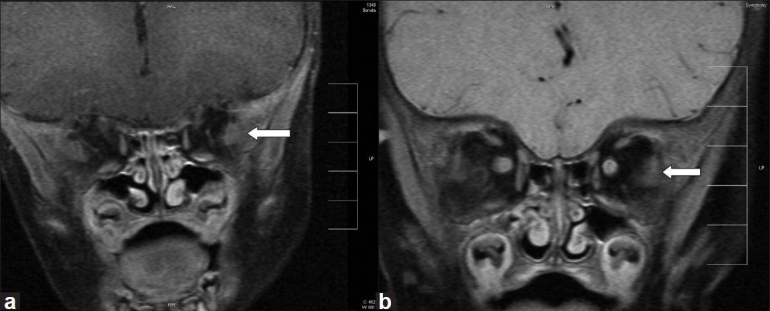
MRI orbits of patients 2 (a) and 3 (b). T1-weighted images showing remarkable atrophy of all extraocular muscles except lateral rectus (white arrow)

All the patients had an abnormal head posture (mainly chin up), a lack of facial expression, and complete to severe bilateral ptosis. Two of three patients demonstrated a positive jaw-winking phenomenon. Mild to moderate visual impairment secondary to ptosis and astigmatism was noted in all of them. Orthoptic examination showed moderate to large angle exotropia with varying amount of hypotropia and limitations of almost all the extra ocular muscles, with relative sparing of the lateral recti [Figures 1 and 2]. All patients demonstrated pupillary abnormalities. Based on the findings of clinical examinations all patients were diagnosed by us as CFEOM type II. The clinical characteristics and the findings of magnetic resonance imaging (MRI) scans of the brain and orbit are summarized in Table 1, and Figures 3 and 4.[3–6]

All the patients received optimal refractive correction and are under follow-up.

**Table 1:** Clinical assessment

	*Patient details*	*General impression*	*Strabismus*	*Ocular motility*	*Visual acuity*	*Pupils*
Patient 1	Omani female 10 years*	AHP: Chin up Ptosis: OU near complete ptosis with scar from previous surgery (frontalis suspension) LPS action: 0–1mm No jaw-winking phenomena No Bell’s phenomena Margin Reflex Distance OU = −2mm	Hypotropia Large Right XT	OD: IOUA −4, SRUA −4, MRUA −1 down shoot in adduction. OS: IOUA −4, SRUA −4, IRUA −4, LRUA −3, MRUA −3	1.0; 0.63	Pupils: OU: 3mm irregular, sluggish reaction
Patient 2	Omani male 9 month*	AHP: Chin up Ptosis: OU Severe ptosis OD>OS. LPS action minimal OU. Lagophthalmos OU Positive jaw-winking phenomena Margin Reflex Distance = OD: 0.5mm; OS: 3 mm	Hypotropia OD Large Left XT	OU: IOUA −4, SRUA −4, LRUA −1.5, some restriction of the adduction.	OU – FF, CSM	Pupils - equal in size and shape, sluggish reaction
Patient 3	Omani male 7 years*	AHP: Chin up, face turned to the left OU Severe ptosis OD>OS. LPS action minimal OU. Lagophthalmos OU Positive jaw-winking phenomena Margin Reflex Distance = OD: 0.50 mm; OS: 3.00 mm	Hypotropia OD Large Right XT	OU: MRUA −4, SRUA −4, IOOA −4, SOUA −4, IRUA −4, LRUA −0.50.	OU – 0.5	Pupils: OU:3mm sluggish reaction

* - Age at presentation; AHP – Abnormal head posture; CSF – Cerebrospinal fluid; EOM – Extraocular muscles; FF – Fixates and follows; CSM – Central, steady and maintained fixation; Motility IOUA – Inferior oblique underaction; SOUA – Superior oblique underaction; SRUA – Superior rectus underaction; IRUA – Inferior rectus underaction; MRUA – Medial rectus underaction; LRUA – Lateral rectus underaction; OD – Right eye; OS – Left eye; OU – Both eyes; XT – Exotropia

## Discussion

CFEOM is a rare, congenital, and non progressive disorder with multiple extra ocular muscle restrictions. Its diagnosis and classification is defined by clinical characteristics and genetics. Based on clinical features and genetics, CFEOM can be classified into three types [Table 2]. Bilateral cases of CFEOM might be very asymmetrical. Numerous ocular and systemic associations have been described in patients with CFEOM [Table 3].[6] CFEOM has to be differentiated from other conditions which might mimic it [Table 4].[6] The diagnosis of CFEOM is made by combining the findings of clinical examination, forced duction test, radiological investigations and genetic analysis. This approach affords the best results in planning management. All our patients received the diagnosis of CFEOM type II. However, due to the overlap in the clinical features between different CFEOM groups, genetic evaluation is important in confirming the diagnosis.[7]

**Table 2:** Classification of CFEOM[1–5]

*Type 1*	*Type 2*	*Type 3 (A, B, C)*
Orthoptics:		
Bilateral ptosis Hypotropia Restricted upgaze, Horizontal strabismus is common, variable restricted horizontal gaze In addition, pupils are often small and non-reactive Positive forced duction	Bilateral ptosis Exotropia Severe restriction of the horizontal and vertical eye movements, variable abduction is present Miotic, poorly reactive pupils Positive forced duction	Some affected individuals do not have classic findings of the disorder. Their eyes may not be infraducted or may elevate above the midline The eyes may be unilaterally affected Ptosis may be absent or variable Positive forced duction
Pathogenesis:		
Absence of the superior division of the oculomotor nerve.	Absence of the motor neurons in all of the oculomotor and trochlear nuclei with abnormalities of the innervated muscles	Variable developmental anomaly of the oculomotor nerve, (superior branch > inferior branch)
Abnormalities of the levator palpebrae superior and rectus superior		
Genetics:		
Locus chromosome 12	Locus – chromosome 11	A: Locus – Chromosome 16
Gene – *KIF21A*	Gene – *PHOX2A (ARIX/11q13)*	Gene – *TUBB3*
Autosomal dominant	Autosomal recessive	B: Locus chromosome 12
Fully penetrant		Gene – *KIF21A*
Variable expression		C: Locus – Chromosome 13
		Gene – unknown
		Autosomal dominant
		Incomplete penetrance
		Variable expression

**Table 3:** Ocular and systemic associations[4]

Ocular associations CFEOM
Refractive errors / amblyopia Neural misdirection – MG phen., synergistic divergence / convergence Optic nerve dysplasia or hypoplasia Chorioretinal coloboma Microphthalmia Oculocutaneous albinism Marcus Gunn jaw – winking phenomenon
Systemic associations CFEOM
Other cranial N anomalies – V, VII Facial dysmorphism Neurodevelopmental defects

**Table 4:** Differential diagnoses of CFEOM[3]

Neurogenic
Congenital III nerve palsy Partial or complete VI nerve palsy Chronic progressive external ophthalmoplegia
Restrictive
Brown’s syndrome Orbital floor fracture Thyroid eye disease Double elevator palsy Möbius’ syndrome Atypical Duane Syndrome
Myogenic with systemic involvement
Myastenia gravis Kearns-Sayre Syndrome

CFEOM can be associated with neuro- radiological abnormalities, and neuroimaging has been recommended as part of the evaluation of patients with CFEOM to rule out any intracranial or orbital pathology.[2] Unilateral or bilateral hypoplasia of CN 3 has been demonstrated using high-resolution MRI in many cases of CFEOM.[8] Hypoplasia of CN 3 supports a neuropathic rather than myopathic origin of CFEOM.

CFEOM is not easy to treat. Any refractive error and amblyopia should be corrected. Due to the extreme chin up posture adopted by some of the patients with CFEOM, eccentric viewing through the corrective lenses is commonly encountered, contributing to a sub optimal refractive correction. This might underlie the reduced visual acuity seen in our patients. Significant changes have been observed in refraction following extraocular muscle surgery secondary to a change in magnitude and direction of the force exerted by the muscles on the globe.[9]

The surgical correction of strabismus and ptosis in CFEOM is challenging. Strabismus surgery is always attempted before ptosis correction. The expectations of strabismus surgery should be realistic and parents and patient should be well informed about these expectations. Very large recessions (12mm) of the affected muscles may be indicated. In CCDDs, resections of extraocular muscles are usually avoided from fear of worsening the enophthalmos.[6] A forced duction test should be done pre-operatively and during the strabismus surgery. With respect to ptosis surgery, due to the absence of Bell’s phenomenon and the risk of exposure keratopathy, it is advisable that ptosis is under-corrected. The aim of ptosis correction should be to provide a clear visual axis, partly eliminate the head posture, and prevent deprivation amblyopia.[10]

In conclusion, CFEOM is a rare, congenital, and non-progressive disorder with multiple extra ocular muscle restrictions. CFEOM can be associated with neuro- radiological abnormalities; its diagnosis and classification is defined by clinical characteristics and genetics. Options for treatment are limited and difficult, and results of surgery are unpredictable.
